# Applicability of the REDUCE‐IT trial to the FAST‐MI registry. Are the results of randomized trials relevant in routine clinical practice?

**DOI:** 10.1002/clc.23437

**Published:** 2020-07-28

**Authors:** Jean Ferrières, Vincent Bataille, Etienne Puymirat, François Schiele, Tabassome Simon, Nicolas Danchin, for the FAST‐MI investigators

**Affiliations:** ^1^ Department of Cardiology Toulouse Rangueil University Hospital Toulouse University School of Medicine Toulouse France; ^2^ Department of Epidemiology and Public Health UMR 1027, INSERM–Université de Toulouse Toulouse France; ^3^ Assistance Publique‐Hôpitaux de Paris (AP‐HP), Department of Cardiology Hôpital Européen Georges Pompidou Paris France; ^4^ Université Paris‐Descartes Paris France; ^5^ INSERM U‐970 Paris France; ^6^ Department of Cardiology Regional University Hospital Jean Minjoz Besançon France; ^7^ Department of Clinical Pharmacology and Unité de Recherche Clinique (URCEST) AP‐HP, Hôpital Saint Antoine Paris France; ^8^ Université Pierre et Marie Curie (UPMC‐Paris 06) Paris France; ^9^ INSERM U‐698 Paris France

**Keywords:** eicosapentaenoic acid, omega‐3 icosapent ethyl, randomized trial, registry, statins

## Abstract

**Background:**

The reduction of cardiovascular events with icosapent ethyl‐intervention trial (REDUCE‐IT) trial revealed robust atherosclerotic cardiovascular risk reduction with a strategy comprising high‐dose omega‐3 icosapent ethyl vs placebo in statin‐treated patients with elevated triglycerides and controlled low‐density lipoprotein cholesterol (LDL‐C).

**Hypothesis:**

Are the results of the REDUCE‐IT trial applicable to the French registry on acute ST‐elevation and non‐ST‐elevation myocardial infarction (FAST‐MI) population?

**Methods:**

Data were extracted from the FAST‐MI 2010 and 2015 registries. We applied the REDUCE‐IT enrolment criteria (triglycerides 150‐500 mg/dL and LDL‐C 40‐100 mg/dL on statins) to the FAST‐MI population in patients aged ≥45 years who had detailed lipid values postacute hospitalization, focusing on their clinical profile and cardiovascular prognosis.

**Results:**

Of the 3789 FAST‐MI patients with a full lipid profile (median 11.1 [IQR 7.6‐17.4] months after hospitalization for myocardial infarction), 472 (12.5%; 95% CI 11.4‐13.5) met the eligibility criteria for REDUCE‐IT (REDUCE‐IT‐like group). The cardiovascular event rate (all‐cause death, nonfatal myocardial infarction, nonfatal stroke) was 36.7 (95% CI 27.8‐48.6) per 1000 person‐years for the REDUCE‐IT‐like group, which compares with the 36.9 (95% CI 26.1‐51.5) per 1000 person‐years (cardiovascular death, nonfatal myocardial infarction, nonfatal stroke) reported in the REDUCE‐IT trial. The residual cardiovascular risk related to elevated triglycerides in the REDUCE‐IT‐like group was similar to the risk in the REDUCE‐IT trial.

**Conclusions:**

If the results of REDUCE‐IT are applied to patients hospitalized for a myocardial infarction in France, 12.5% of these patients could benefit from a strategy of high‐dose omega‐3 icosapent ethyl on top of contemporary therapy including statins to improve their clinical outcomes.

## 1 INTRODUCTION

Cardiovascular disease (CVD), remains the most common cause of death worldwide.^1^ High‐intensity statin treatment reduces the risk of Atheroclerotic Cardiovascular Disease (ASCVD) events in both primary and secondary prevention,^2^ but substantial residual risk remains even after significant reductions in low‐density lipoprotein cholesterol (LDL‐C) with the use of intensive lipid‐lowering therapies (LLT).^3^, ^4^, ^5^, ^6^ In such patients, elevated triglycerides are regarded as an additional independent risk factor for ischemic events,^7^, ^8^, ^9^, ^10^ and represent a target for investigation with triglyceride‐lowering medications. Randomized trials with extended‐release niacin^11^ and fibrates,^12^, ^13^, ^14^ which reduce triglyceride levels and raise high‐density lipoprotein levels, have failed to demonstrate consistent cardiovascular event reduction on a background of statin therapy, whereas two studies of eicosapentaenoic acid (EPA) showed a reduction in the risk of cardiovascular events in statin‐treated patients with raised triglycerides.^15^, ^16^ The reduction of cardiovascular events with icosapent ethyl‐intervention trial (REDUCE‐IT) trial^16^ involved 8179 patients with established CVD or with diabetes and other risk factors who had been receiving statin therapy and had a fasting triglycerides level of 135‐499 mg/dL and an LDL‐C level of 41‐100 mg/dL. Patients were randomly assigned to receive icosapent ethyl 4 g/d or placebo. Icosapent ethyl reduced the risk of the primary endpoint (a composite of cardiovascular death, nonfatal myocardial infarction, nonfatal stroke, coronary revascularization, or unstable angina) by 25% (hazard ratio [HR] 0.75; 95% confidence interval [CI] 0.68‐0.83; *P* < .001) and the key secondary endpoint of cardiovascular death, nonfatal myocardial infarction, or nonfatal stroke by 26% (HR 0.74; 95% CI 0.65‐0.83; P < .001). However, patients on icosapent ethyl were more likely to be hospitalized for atrial fibrillation or flutter (*P* = .004). The REDUCE‐IT trial confirmed that raised baseline triglycerides in patients with well‐controlled LDL‐C on statins is a major contributor to ASCVD risk.^16^ One of the limitations of randomized trials is that their populations may not be representative of those treated in routine clinical practice.^17^ We therefore sought to evaluate the applicability of the REDUCE‐IT results to an unselected French population with a previous myocardial infarction, by applying the eligibility criteria for REDUCE‐IT to the French registry on acute ST‐elevation and non‐ST‐elevation myocardial infarction (FAST‐MI) 2010 and 2015 registries.

## 2 METHODS

The primary objectives of the nationwide FAST‐MI 2010^18^ and 2015^19^ registries were to evaluate the characteristics, management, and outcomes of patients with acute myocardial infarction (AMI) treated in routine clinical practice in France.^18^, ^19^, ^20^ Based on the eligibility criteria for the REDUCE‐IT trial,^16^ we identified patients in the FAST‐MI 2010 and 2015 registries who were aged 45 years or older, had triglyceride levels between 150 and 500 mg/dL, LDL‐C levels of 40‐100 mg/dL while on statin therapy, and had detailed lipid values available postacute hospitalization (ie, at the start of follow‐up). These patients were classified into two groups: “REDUCE‐IT‐like” (those who fulfilled the REDUCE‐IT trial inclusion criteria and had no exclusion criteria); and “REDUCE‐IT‐excluded” (those who had at least 1 REDUCE‐IT exclusion criterion). To be consistent with REDUCE‐IT and reflect a population with chronic coronary heart disease (CHD), this analysis included lipid‐lowering and other drugs taken after a period of stabilization following the index event.

Clinical outcomes for FAST‐MI were the composite of a major adverse cardiovascular events (MACE: all‐cause death, nonfatal myocardial infarction, or nonfatal stroke), and the individual components of the composite outcome. In the REDUCE‐IT trial, cardiovascular death (rather than all‐cause death) was used in the composite (key secondary) MACE outcome. We included all‐cause death because information on cardiovascular death, as reported in REDUCE‐IT, was not collected in the FAST‐MI registries.

Continuous variables are reported as means ± SDs or medians with interquartile ranges (IQRs), as appropriate. Discrete variables are described as counts and percentages. REDUCE‐IT‐like and REDUCE‐IT‐excluded groups were compared by analysis of variance for continuous variables, and by the χ^2^ test (or Fisher's exact test) for discrete variables. Statistical analyses were performed using StataCorp 2007 (Stata Statistical Software: Release 10, College Station, TX: StataCorp LP). For all analyses, two‐sided *P* values <.05 were considered significant.

## 3 RESULTS

Overall, 9459 patients were included in the FAST‐MI 2010 and 2015 registries, of which 3789 had a full lipid profile available after a period of stabilization (median of 11.1 [IQR 7.6‐17.4] months after hospitalization for AMI) (ie, the start of follow‐up). Of these patients, 472 (12.5%, 95% CI 11.4‐13.5) met the enrolment criteria for the REDUCE‐IT trial. The baseline characteristics (ie, at the time of hospitalization for the index event) for the overall population and for REDUCE‐IT‐like and REDUCE‐IT‐excluded patients are shown in Table 1. Compared with REDUCE‐IT Excluded patients, REDUCE‐IT‐like patients were less frequently aged ≥65 years and more frequently male, obese, smokers, and have hypertension or diabetes.

**TABLE 1:** Baseline characteristics (during hospitalization for index acute myocardial infarction) of patients enrolled in the FAST‐MI (2010 and 2015) registries

Characteristic	All FAST‐MI patients (n = 3789)	REDUCE‐IT‐Excluded (n = 3317)	REDUCE‐IT‐Like (n = 472)	*P* value
Clinical characteristics	–	–	–	–
Men	2822 (74.5%)	2447 (73.8%)	375 (79.4%)	.008
Age (years)	64 (55‐74)	65 (55‐74)	61 (54‐69)	.14
Age ≥ 65 y	1848 (48.8%)	1666 (50.2%)	182 (38.6%)	<.001
Qualifying event (for inclusion)	–	–	–	.80
NSTEMI	1753 (46.3%)	1532 (46.2%)	221 (46.8%)	–
STEMI	2036 (53.7%)	1785 (53.8%)	251 (53.2%)	–
Risk factors, n (%)	–	–	–	–
Arterial hypertension	1926/3778 (51.0%)	1660/3307 (50.2%)	266/471 (56.5%)	.011
BMI ≥30 kg/m^2^	776/3648 (21.3%)	622/3190 (19.5%)	154/458 (33.6%)	<.001
Diabetes	653/3773 (17.3%)	514/3301 (15.6%)	139/472 (29.4%)	<.001
Current smoker	1209/3653 (33.1%)	1000/3189 (31.4%)	209/464 (45.0%)	<.001
Presenting characteristics	–	–	–	–
Killip class II − IV	342/3513 (9.7%)	290/3076 (9.4%)	52/437 (11.9%)	.103
Killip class III or IV	139/3513 (4.0%)	115/3076 (3.7%)	24/437 (5.5%)	.08
Last LVEF during initial hospitalization (%)	(n = 2861)	(n = 2502)	(n = 359)	–
Mean ± SD	53.0 ± 10.0	53.1 ± 10.0	52.3 ± 9.7	.17
Median (IQR)	55 (45‐60)	55 (45–60)	53 (45–60)	.16

*Note:* Data given as number (%), n/N (%), mean ± SD or median (IQR).

Abbreviations: FAST‐MI, French registry on acute ST‐elevation and non‐ST‐elevation myocardial infarction; IQR, interquartile ranges; REDUCE‐IT, reduction of cardiovascular events with icosapent ethyl‐intervention trial.

Cardiovascular secondary prevention medications being taken during the chronic phase, along with the patients' lipid levels, are detailed in Table 2. During the chronic phase, 97.7% of the REDUCE‐IT‐like patients were on moderate‐ or high‐intensity statin therapy and 8.7% were on ezetimibe; the mean ± SD LDL‐C level was 72 ± 16 mg/dL (Table 2). Median triglyceride levels were 192 mg/dL, and 16.1% had a combination of triglycerides >200 mg/dL and HDL‐C < 35 mg/dL. Over half (n = 261, 55.3%) of the population had triglyceride levels of 150‐199 mg/dL, 36.0% (n = 170) had levels of 200‐299 mg/dL, and 8.7% (n = 41) had levels of 300‐500 mg/dL.

**TABLE 2:** Secondary prevention medications and lipid levels during the chronic phase (median 11.1 months after the index event)

	All patients (n = 3789)	REDUCE‐IT‐Excluded (n = 3317)	REDUCE‐IT‐Like (n = 472)	*P* value
Selected cardiac medications				
Statin	3314 (87.5%)	2842 (85.7%)	472 (100.0%)	–
Statin intensity among those on a known‐dose statin	–	–	–	0.03
Low dose^a^	80/3309 (2.4%)	69/2837 (2.4%)	11/472 (2.3%)	–
Moderate dose^b^	898/3309 (27.1%)	746/2837 (26.3%)	152/472 (32.2%)	–
High dose^c^	2331/3309 (70.4%)	2022/2837 (71.3%)	309/472 (65.5%)	–
Ezetimibe	210 (5.5%)	169 (5.1%)	41 (8.7%)	.001
Beta‐blocker	3065 (80.9%)	2656 (80.1%)	409 (86.7%)	.001
ACE inhibitor or angiotensin II receptor blocker	3366 (88.8%)	2923 (88.1%)	443 (93.9%)	<.001
Antiplatelet	3585 (94.6%)	3130 (94.4%)	455 (96.4%)	.07
Lipid values (mg/dL)				
Triglycerides, median (IQR)	104 (77‐143)	96 (74‐125)	192 (166‐235)	<.001
Total cholesterol, mean ± SD	160 ± 53	161 ± 54	159 ± 41	.43
HDL‐C, mean ± SD	52 ± 27	53 ± 27	43 ± 23	<.001
LDL‐C, mean ± SD	(n = 3728) 85 ± 37	(n = 3272) 86 ± 39	(n = 456) 72 ± 16	<.001
Triglycerides >200 mg/dL + HDL‐C < 35 mg/dL	147 (3.9%)	71 (2.1%)	76 (16.1%)	<.001

*Note:* Data given as number (%), n/N (%), mean ± SD or median (IQR).

Abbreviations: HDL‐C, high‐density lipoprotein cholesterol; LDL‐C, low‐density lipoprotein cholesterol; REDUCE‐IT, reduction of cardiovascular events with icosapent ethyl‐intervention trial.

^a^ Low dose: simvastatin 10 mg/d, pravastatin 10‐20 mg/d, or fluvastatin 20‐40 mg/d.

^b^ Moderate dose: atorvastatin 10‐20 mg/d, rosuvastatin 5‐10 mg/d, simvastatin 20‐40 mg/d, pravastatin 40 mg/d, or fluvastatin 80 mg/d.

^c^ High dose: atorvastatin 40‐80 mg/d or rosuvastatin 20‐40 mg/d.

The MACE rate was 36.7 (95% CI 27.8‐48.6) per 1000 patient‐years for REDUCE‐IT‐like patients and 31.1 (95% CI 27.8‐34.8) per 1000 patient‐years for REDUCE‐IT‐excluded patients, which compares with 36.9 (95% CI 26.1‐51.0) per 1000 patient‐years for 5785 patients with established CVD in the REDUCE‐IT trial (reported in the US Food and Drug Administration briefing document^21^) (Table 3). The rate of atrial fibrillation or flutter requiring hospitalization for ≥24 hours was 1.5 (95% CI 0.4‐6.0) per 1000 patient‐years for the REDUCE‐IT‐like patients and 6.0 (95% CI 4.6‐7.7) per 1000 patient‐years for REDUCE‐IT‐excluded patients. The REDUCE‐IT trial reported that 2.2% of placebo‐treated patients in the secondary prevention subgroup developed atrial fibrillation or flutter.^21^

**TABLE 3:** Event rates in the REDUCE‐IT trial^21^ and the REDUCE‐IT‐Like and REDUCE‐IT‐Excluded subgroups in the FAST‐MI 2010 and 2015 registries

Outcome, per 1000 patient‐years (95% CI)	REDUCE‐IT trial patients		FAST‐MI 2010 + 2015	
	Established CVD (n = 5785)	Diabetes mellitus + risk factor for CVD (n = 2394)	REDUCE‐IT‐Excluded (n = 3317)	REDUCE‐IT‐Like (n = 472)
Composite outcome	36.9 (26.1‐51.0)^a^	20.7 (13.0‐32.1)^a^	31.1 (27.8‐34.8)^b^	36.7 (27.8‐48.6)^b^
Total death	18.7 (11.4‐29.7)	12.1 (6.2‐21.0)	19.4 (17.0‐22.1)	24.3 (17.7‐33.4)
Nonfatal myocardial infarction	20.4 (12.2‐30.9)	8.8 (4.1‐17.1)	6.9 (5.4‐8.7)	9.1 (5.2‐16.0)
Nonfatal stroke	6.3 (2.2‐13.1)	4.9 (1.6‐11.7)	2.8 (1.9‐4.1)	2.2 (0.7‐7.0)

Abbreviations: CI, confidence interval; CVD, cardiovascular disease; FAST‐MI, French registry on acute ST‐elevation and non‐ST‐elevation myocardial infarction; REDUCE‐IT, reduction of cardiovascular events with icosapent ethyl‐intervention trial.

^a^ Cardiovascular death, nonfatal myocardial infarction, or nonfatal stroke for REDUCE‐IT.

^b^ All‐cause death, nonfatal myocardial infarction, or nonfatal stroke for FAST‐MI.

## 4 DISCUSSION

By applying the enrolment criteria of the REDUCE‐IT trial to patients in the FAST‐MI 2010 and 2015 registries, 12.5% of this nationwide French population would have been eligible for enrolment. Given the 26% reduction in the key secondary outcome of cardiovascular death, nonfatal myocardial infarction or nonfatal stroke with the use of high‐dose icosapent ethyl vs placebo in REDUCE‐IT,^16^ these French patients could benefit from treatment with EPA on top of contemporary medical therapy including statins to improve their future cardiovascular health.

REDUCE‐IT‐like patients in FAST‐MI exhibited a slightly different clinical profile to patients with established CVD in the REDUCE‐IT trial.^21^ All of the patients in FAST‐MI had a history of ASCVD, compared with 70.7% in REDUCE‐IT. The median age of REDUCE‐IT‐like patients was 61 years, compared with 64 years in REDUCE‐IT, and a high percentage (45.0%) were current smokers (at the time of the index event) compared with 16.5% in REDUCE‐IT; this difference in smoking prevalence likely reflects the acute stage of their disease before secondary prevention measures and lifestyle recommendations had been adopted. REDUCE‐IT‐like patients also had lower rates of hypertension (56.5% vs 97.8%) and diabetes (29.4% vs 41.2%). The overall rates of moderate‐ or high‐intensity statin therapy were similar (97.7% in REDUCE‐IT‐like patients vs 95.5% in REDUCE‐IT), but the proportion of patients on high‐intensity statin therapy was greater in the REDUCE‐IT‐like population (65.3% vs 35.2%). Regardless of these differences, the residual cardiovascular risk related to elevated triglycerides in the REDUCE‐IT‐like population was similar, with an overall cardiovascular event rate of 36.7 per 1000 patient‐years, compared with 36.9 per 1000 persons‐years in patients with established CVD in REDUCE‐IT. However, the composite outcome included all‐cause death in the REDUCE‐IT‐like population and only cardiovascular death in the REDUCE‐IT trial. Whereas the rate of all‐cause death was higher in the REDUCE‐IT‐like population, the rates of nonfatal myocardial infarction or stroke were lower. These differences may reflect lower rates of CVD in France or a higher risk of atherosclerosis or other coexisting conditions (such as diabetes and hypertension) in REDUCE‐IT.

Several studies have applied the REDUCE‐IT enrolment criteria to their study populations.^22^, ^23^, ^24^, ^25^ In the Veterans Affairs healthcare system,^24^ 14.5% of the population with ASCVD (defined as a history of myocardial infarction, ischemic stroke, or peripheral artery disease) would have met the trial criteria. However, the use of moderate‐ to high‐intensity statin was lower in the Veterans Affairs population (approximately 80%, compared with 95.5% in patients with ASCVD in REDUCE‐IT), suggesting that the size of the eligible population could decrease with intensification of statin treatment (leading to a reduction in median triglyceride levels). In the CLARIFY registry,^22^ which involved patients with stable coronary artery disease (defined as a history of myocardial infarction, coronary stenosis >50%, symptomatic myocardial ischemia, or previous coronary revascularization procedure), 15.5% would have broadly met the enrolment criteria for REDUCE‐IT. One‐quarter of the ONTARIO study^23^ population were reported to be eligible, but the population was broad based and included patients with a history of myocardial infarction, ischemic stroke, peripheral artery disease, or previous coronary revascularization procedure. The most similar study to FAST‐MI was a population‐based Danish cohort study in patients with myocardial infarction,^25^ in which 21% met the enrolment criteria for REDUCE‐IT, substantially more than those in the FAST‐MI registries. The Danish population may have had a higher prevalence of hypertriglyceridaemia, as suggested by the median triglyceride value of 214 (177‐277) mg/dL compared with 192 (166‐235) mg/dL in the REDUCE‐IT‐like population.

Our study has several strengths and limitations. Based on the information available in FAST‐MI, we were able to select patients who would have fulfilled all of the REDUCE‐IT study criteria. FAST‐MI was restricted to patients hospitalized with AMI rather than all patients with established CVD. We collected all‐cause death and not cardiovascular death in the FAST‐MI registries; therefore, the contribution of death to the composite MACE outcome in the FAST‐MI population will be greater than that of cardiovascular death in the REDUCE‐IT trial. However, all this information is available in the REDUCE‐IT trial and therefore, the comparison with the REDUCE‐IT‐Like population will be possible as soon as the results will be published by the REDUCE‐IT authors.

## 5 CONCLUSIONS

The residual cardiovascular risk related to elevated triglycerides in the REDUCE‐IT‐like group was similar to the risk in the REDUCE‐IT trial. If the results of REDUCE‐IT are applied to patients hospitalized for a myocardial infarction in France, 12.5% of these patients could benefit from a strategy of high‐dose omega‐3 icosapent ethyl on top of contemporary therapy including statins to improve their clinical outcomes.

## DISCLOSURE OF INTERESTS

J. F. reports grants and personal fees from Akcea, Amarin, Amgen, MSD, Sanofi and Servier. V. B. has nothing to disclose. E. P. reports fees for lectures and/or consulting from Amgen, AstraZeneca, Bayer, Biotronik, BMS, Boehringer Ingelheim, Daiichi‐Sankyo, Lilly, MSD, The Medicine Company, Sanofi, Saint Jude Medical, Servier, Siemens. F. S. reports personal fees from Amgen, Astra Zeneca, Bayer, BMS, MSD, Pfizer, and Sanofi. T. S. reports grants from AstraZeneca, Daiichi‐Sankyo, Eli‐Lilly, GSK, MSD, Novartis, Sanofi, and personal fees for board membership and/or consultancy and/or lectures from AstraZeneca, BMS, Sanofi, and Novartis. N. D. reports having received grants, speaking fees, consulting fees, or nonfinancial support from Amgen, AstraZeneca, Bayer, BMS, Boehringer‐Ingelheim, Intercept, Novo‐Nordisk, Pfizer, Sanofi and Servier.
